# Radiation recall pneumonitis in the setting of immunotherapy and radiation: a focused review

**DOI:** 10.2144/fsoa-2018-0123

**Published:** 2019-04-10

**Authors:** Kerri McGovern, Maged Ghaly, Michael Esposito, Keara Barnaby, Nagashree Seetharamu

**Affiliations:** 1 Division of Hematology & Medical Oncology, Donald & Barbara Zucker School of Medicine at Hofstra/Northwell, Lake Success, NY, 11042 USA; 2 Department of Radiation Oncology, Donald & Barbara Zucker School of Medicine at Hofstra/Northwell, Lake Success, NY, 11042 USA; 3 Department of Pathology, Donald & Barbara Zucker School of Medicine at Hofstra/Northwell, Lake Success, NY, 11042 USA

**Keywords:** checkpoint inhibitors, immunotherapy, pneumonitis, radiation recall pneumonitis, thoracic radiation

## Abstract

Radiation recall pneumonitis (RRP) is an entity described as pneumonitis localized to a previously irradiated field after exposure to a systemic agent. It has previously been described in the literature in the context of chemotherapeutic agents as well as certain biologics. With immunotherapy taking a more prominent role in the treatment of several different malignancies and its own baseline risk of pneumonitis, it is important to explore the likelihood of RRP, specifically in those patients who have been previously treated with radiation therapy. The current literature regarding RRP with checkpoint inhibitors is reviewed in this article. Alongside this review, we report a case of RRP after pembrolizumab initiation in a patient in our practice.

## Lay abstract

When patients undergo radiation to the chest there is a risk of inflammation to the lung tissue called pneumonitis. This inflammation can also be a result of certain medications, including checkpoint inhibitors, which are used to treat cancers. There is a specific phenomenon, radiation recall pneumonitis, where patients who receive radiation to the chest followed by checkpoint inhibitors experience pneumonitis only in the lung fields that were exposed to previous radiation. This paper reviews the existing body of literature and describes a case of radiation recall pneumonitis seen in our practice.

Radiation recall is an unpredictable, poorly understood entity wherein an acute inflammatory response confined to a previously irradiated area ensues upon exposure to a systemic agent. Most of the cases of radiation recall reported in the literature are cutaneous, but a handful of reports pertaining to other sites have been reported. In this report, we focus on radiation recall pneumonitis (RRP), which is considered a type of radiation-related lung injury that presents as acute inflammation limited to previously irradiated lung fields, weeks, months or even years after completion of radiation therapy in response to a systemic trigger. Many systemic agents have been reported to trigger RRP but to our knowledge, there is very limited literature on RRP induced by checkpoint inhibitors. In this manuscript, we report a case of PD-1 inhibitor-associated RRP and present a focused review on the topic.

## Etiopathogenesis of RRP

The mechanistic details of RRP are not completely characterized. However, several mechanisms have been postulated [1]; the foremost of them is that the stem cells in irradiated tissue are subject to sublethal damage resulting in aberrant function including their ability to proliferate. Subsequent generations of cells in that same irradiated tissue become more sensitive and mount an inflammatory response in the presence of a systemic toxin. It has also been postulated that radiation therapy may induce hypersensitivity to certain cytotoxic agents. Last, the pharmacodynamics of the systemic agent may be altered by radiation, resulting in elevated concentration of these agents in previously irradiated sites. It is interesting to note that almost all systemic agents that have been reported to induce RRP have also been shown to result in pulmonary toxicity in the form of interstitial pneumonitis and pulmonary fibrosis independent of radiation. Hence, one might theorize that synergistic damage to type II pneumocytes from specific systemic agents and radiation may lead to RRP [1].

## Systemic agents associated with RRP

RRP has been described in the literature in association with several classical chemotherapeutic agents such as paclitaxel [2] and gemcitabine [3]. With the evolution of cancer care using biological and immunological therapies, which are themselves associated with pulmonary toxicities, there seems to be a renewed interest in RRP as an entity. There are recent reports of RRP in patients treated with small-molecule tyrosine kinase inhibitors such as the targeted agents everolimus and sunitinib [4,5]. Recently, a small number of cases of RRP induced by checkpoint inhibitor therapy were reported in the literature [6]. In this manuscript, we report a case of PD-1 inhibitor-associated RRP and present a focused review on the topic.

## Chemotherapeutic agents

RRP has been described in association with several classical chemotherapeutic agents. Azria *et al*. performed a Medline and Cancerlit search for radiation recall in 2005 and reported taxanes and anthracyclines to be responsible for 20 and 30% of radiation recall reactions, respectively [7]. Subsequently, Ding *et al*. published the largest case series of RRP, involving 12 patients [8]. They reported that 8 of the 12 patients in their series had been exposed to taxanes. Other chemotherapeutic agents implicated in RRP include ifosfamide, etoposide, pemetrexed and platinum [9–11].

## Biological and other agents

Several small-molecule kinase inhibitors, particularly those inhibiting the MAPK and mTOR pathways have been associated with pulmonary toxicity. Some of the agents implicated in causing RRP include erlotinib, gefitinib, afatinib, sunitinib and everolimus [12]. These same agents are also linked to RRP in recently published case reports. In 2016, Chiang *et al*. reported a 4.4% incidence of RRP in patients with history of prior thoracic receiving EGF tyrosine kinase inhibitor therapy [13]. There are reports of radiation recall with hormonal agents such as tamoxifen as well, though more commonly involving as compared with the lungs [14]. Even nononcological agents such as anti-tuberculosis drugs, statins, antibiotics and antifungal drugs have been reported to cause RRP [15].

## A focus on checkpoint inhibitor therapy

Antibody blockade of the programmed death (PD-1) molecule or its ligand (PD-L1) have revolutionized treatment for various types of cancers including lung cancer, head and neck cancer, melanoma, renal cell carcinoma and lymphoma. By engaging with PD-L1 expressed on tumor cells, these molecules inhibit the immunosuppression rendered by interaction of PD-L1 with PD-1 on T cells, and restore anti-tumor T cell responses [16]. While these agents have shown anticancer activity in a plethora of cancers, they also frequently cause immune-related adverse events. It has been suggested that the tumor neoantigens and normal tissue antigens cross-react and therefore with the introduction of checkpoint inhibitors normal tissue in the GI tract, liver, skin and endocrine organs can be damaged along with the tumor cells [17]. One of the well-documented and potentially fatal immune-related adverse events associated with checkpoint inhibitors is pulmonary toxicity or pneumonitis, with an estimated incidence up to 10% (any grade) of which 1–2% are Grade 4 or 5 events [18,19]. Higher rates are seen with combination therapy compared with monotherapy. A recent meta-analysis involving 12 Phase II and III clinical trials (a total of 6240 patients) evaluated the relationship between risk of pneumonitis and pneumonitis-related death and PD-1 inhibitor treatment in patients with cancer [20]. This study showed a modest increase in relative risk of developing pulmonary complications in patients receiving checkpoint inhibitors compared with patients on chemotherapy (RR 2.65; p = 0.06). The incidence of all-grade pneumonitis (Grades 1–4) ranged from 1.3 to 5.8% whereas high-grade (grades 3–4) pneumonitis ranged from 0 to 2.6%. The incidence of pneumonitis-related death was extremely low (0–0.6%).

In clinical practice, we see many patients, particularly those with lung cancer, receiving checkpoint inhibitor therapy who have been previously exposed to thoracic radiation. With the PACIFIC trial, which compared a PD-L1 inhibitor, durvalumab, to observation after chemoradiation in Stage III lung cancer patients, showing significantly improved progression-free and overall survival outcomes [21], we expect that this population will increase tremendously in the near future. Additionally, there are many patients on checkpoint inhibitor therapy who are treated with palliative-intent chest radiation. Hence, it is vital to understand the synergy between these two modalities both in terms of efficacy and toxicity.

Radiation by itself has immunomodulatory effects. It can induce tumor cell death and result in release of damage-associated molecular pattern markers that in turn make tumor antigens more accessible for presentation to cytotoxic T cells by antigen-presenting cells [6]. Radiation can cause alterations in the tumor cell and tumor milieu in a manner that enhances the sensitivity of tumor cells to immune-mediated cell death. Interestingly, immune-mediated regression of not only the irradiated tumor, but also of distant tumors outside the radiation field (abscopal effect) have been extensively reported [22,23]. The strategy of combining ionizing radiation with checkpoint inhibitor therapy is based on the premise that radiation causes direct cell death releasing tumor antigens and initiates an immune response that is further augmented by checkpoint inhibitor treatment [5].

While there is a strong rationale and background data to support combining immunotherapy and thoracic radiation, there is also significant concern for overlapping pulmonary toxicity. A subgroup analysis from the KEYNOTE-001 trial noted that patients with locally advanced lung cancer who had previously received thoracic radiation had prolonged progression-free and overall survival with pembrolizumab compared with those who had not [24]. At the same time, pooled data from all pembrolizumab studies indicated that pneumonitis was more frequent in patients with a history of prior thoracic radiation compared with those without that exposure (6 vs 2.6%). It should be noted that the authors of this paper do not specify whether the lung injury was limited to the field of prior irradiation or generalized [25]. In general, cases of pneumonitis associated with immune checkpoint inhibitors have a generalized multilobar distribution. However, the pattern of lung damage with these agents in patients previously exposed to thoracic radiation is not well characterized.

A handful of cases reported in the current literature have described the phenomenon of RRP in patients who received immunotherapy with PD-1 or cytotoxic T cell lymphocyte-associated antigen-4 (CTLA-4) antibodies after prior exposure to thoracic radiation [26,27]. Most of these patients had received radiation for a long time, in some cases several years prior to treatment with checkpoint inhibitor therapy. Even though the presenting symptoms varied widely from completely asymptomatic to severely symptomatic with cough, dyspnea and fever, all these patients demonstrated radiographic findings of ground-glass opacities, diffuse haziness, infiltrates or consolidation limited to the previously irradiated fields [28]. The radiological findings are similar to RRP seen with other agents. Histopathological evaluations of these lesions show inflammatory changes such as congested mucosa with leukocytic infiltration, exudative alveolitis, presence of giant cells, hyperplastic type 2 pneumocytes and fibrosis.

**Figure 1.**
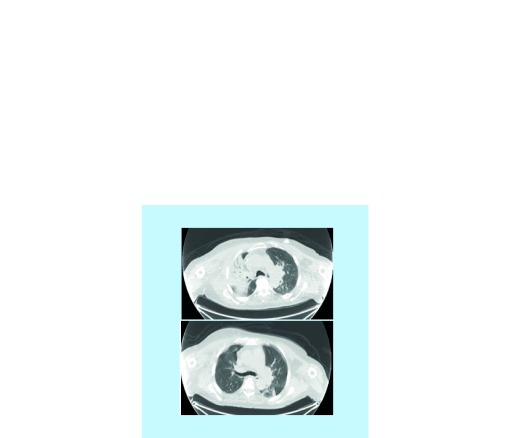
**Top image shows initial right upper lobe dense infiltrate suggestive of radiation pneumonitis and bottom image shows resolution of infiltrate 3 months after initiation of prednisone.**

**Figure 2.**
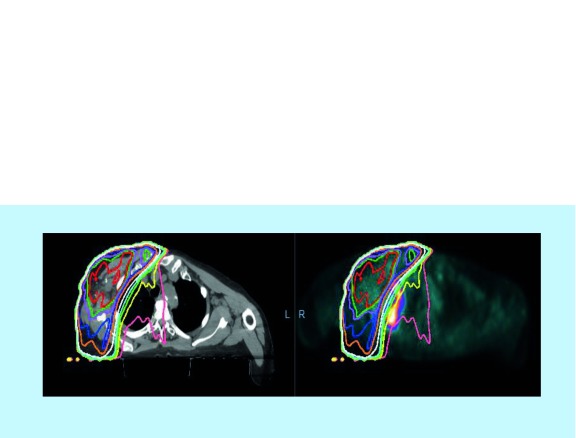
**Prior right lateral chest wall mass radiation dosimetry (right) and radiation field overlying the PET/CT scan visualizing the radiation recall pneumonitis (left).**

**Figure 3.**
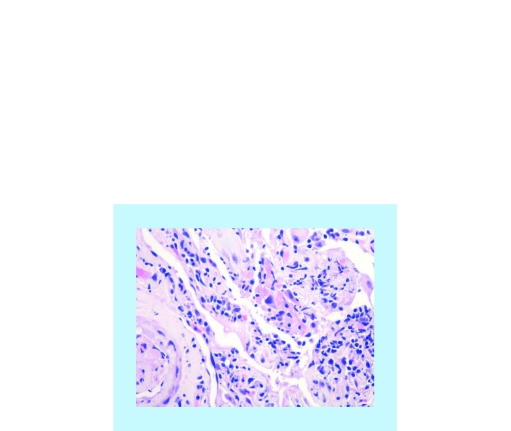
**Chronic inflammation and Type 2 pneumocyte hyperplasia as seen on endobronchial biopsy.**

In our practice, an 82-year-old gentleman with lung adenocarcinoma developed an asymptomatic fluorodeoxyglucose (FDG)-avid right upper infiltrate in October 2017 after receiving pembrolizumab monotherapy starting in December 2016. His history is notable for prior external beam radiotherapy in August 2016 for a right chest wall mass. He was initially treated with antibiotics with no improvement in radiological findings. While the right upper lobe infiltrate was in the previously irradiated field, most of the areas had received low-dose radiation ranging from 5 to 20 Gy (Figures 1 & 2). The patient underwent bronchoscopy with endobronchial biopsy of the lesion and pathology showed chronic inflammation and type 2 pneumocyte hyperplasia (Figure 3). Prednisone 1 mg/kg was initiated followed by a prolonged taper with repeat imaging 3 months later showing resolution of the infiltrate.

## Conclusion

In summary, RRP is a potential risk in patients with prior history of thoracic radiation receiving various systemic agents. This is particularly of concern with checkpoint inhibitor therapies, which are by themselves associated with immune-mediated pneumonitis. Since RRP may mimic cancer progression or pneumonia, there may be significant delay in diagnosis. While there are clear guidelines for immunotherapy-induced pneumonitis, there are none for RRP. Risk for RRP should be borne in mind while starting immunotherapy in patients with history of thoracic radiation and a biopsy should be considered when radiological findings are confined to previously irradiated field(s). As demonstrated by our case, RRP is steroid-sensitive and timely initiation of steroids may result in complete resolution of symptoms.

## Future perspective

Checkpoint inhibitors are a relatively new therapy in the toolbox for oncologists. There is a plethora of ongoing studies looking into the role of checkpoint inhibitors across several different malignancies. With all of these data that are being gathered, there will also be data evolving as to the potential adverse events associated with these medications. Early data have documented some level of risk in terms of RRP, although it will be important to follow these patients over a longer period of time to see if these rates increase. Accurately quantifying the morbidity and mortality associated with RRP may impact how patients are treated in the future, specifically as to where and when steroids are appropriate. Toxicity assessment in future clinical trials should classify RRP as a separate entity and guidelines should be specifically created for this phenomenon.

**Summary points**

- Radiation recall pneumonitis (RRP) is a diagnosis that has been documented with several systemic agents used in the treatment of cancer and it is now starting to be documented with checkpoint inhibitors.
- Both radiation therapy and immune checkpoint inhibitors on their own have immune-mediated adverse effects and therefore the combination of these therapies can lead to synergistic consequences, including pneumonitis.
- There are no specific guidelines for diagnosis and treatment of RRP with treatment extrapolated from immune-mediated pneumonitis secondary to immunotherapy. Moreover, RRP can mimic other lung conditions, which may delay diagnosis. There is a need for trials to specifically evaluate for RRP as an adverse event, which can therefore be utilized to create those guidelines.
